# Appropriate Correction of Hyponatremia in a Patient With Psychogenic Polydipsia: A Case Report

**DOI:** 10.7759/cureus.82147

**Published:** 2025-04-12

**Authors:** Carter R Olberding, Feross Habib, Peter Richa, Ravish Narvel

**Affiliations:** 1 Medicine, Lake Erie College of Osteopathic Medicine, Bradenton, USA; 2 School of Medicine, Lake Erie College of Osteopathic Medicine, Jacksonville, USA; 3 Internal Medicine, Ascension St.Vincent's Riverside, Jacksonville, USA

**Keywords:** central pontine myelinolysis, hyponatremia, osmotic demyelination syndrome, primary polydipsia, psychogenic polydipsia

## Abstract

Hyponatremia, a common electrolyte imbalance characterized by low serum sodium values, can range from mild, asymptomatic cases to life-threatening conditions. Complex etiologies, such as psychiatric disorders, may complicate the presentation and treatment modalities utilized. Additionally, treatment strategies must be personalized, as rapid correction of hyponatremia can lead to severe complications such as osmotic demyelination syndrome (ODS). This case report examines a 69-year-old male with a history of schizophrenia and chronic psychogenic polydipsia who presented with severe hyponatremia, serum sodium 115 mmol/L (millimoles per liter), and associated symptoms. The patient’s condition was complicated by the risk of osmotic demyelination syndrome (ODS) due to the rapid correction of sodium levels. A careful management strategy using dextrose 5% in water (D5W) and desmopressin (DDAVP) was employed to gradually correct his sodium levels and prevent ODS. This case highlights the importance of differentiating between acute and chronic hyponatremia and adhering to correction protocols to avoid dangerous overcorrection. Ultimately, this case reinforces the need for multidisciplinary collaboration, attentive monitoring, and individualized treatment strategies in managing hyponatremia in patients with underlying psychiatric conditions.

## Introduction

Hyponatremia, defined as a serum sodium concentration below 135 mmol/L, is the most prevalent electrolyte disorder encountered in clinical practice, spanning mild asymptomatic cases to severe, life-threatening conditions [1]. Its underlying causes are diverse, encompassing renal, cardiac, hepatic, and endocrine dysfunctions, as well as excessive water intake [1]. Among these primary causes, psychogenic polydipsia stands out as a particularly challenging etiology, frequently linked to psychiatric disorders like schizophrenia, with a prevalence estimated at 6%-20% of all patients with psychiatric diagnoses [2]. Psychogenic polydipsia, characterized by an uncontrollable compulsion to drink excessive amounts of water, overwhelms the kidneys' excretory capacity, leading to dilutional hyponatremia [3]. Despite adequate suppression of antidiuretic hormone (ADH) in these cases, the sheer volume of water intake surpasses the renal capacity to dilute and excrete free water, resulting in dangerously low sodium levels [4]. In fact, symptomatic hyponatremia is only observed once the kidneys are overwhelmed with upwards of 15L/d of water intake [3].

The etiology of psychogenic polydipsia is postulated to be multifactorial; however, one likely cause is the dysregulation of the hypothalamic-pituitary axis secondary to excess water intake [3]. The challenge lies not only in diagnosing psychogenic polydipsia but also in its management, as rapid correction of serum sodium carries the risk of osmotic demyelination syndrome (ODS) and, more specifically, central pontine myelinolysis (CPM) [5]. CPM is a devastating complication that occurs when a rapid increase in serum sodium concentration draws water out of brain cells, leading to osmotic injury and demyelination of neurons, particularly in the pons [5]. The neurological sequelae of hyponatremia are broad ranging, from mild to severe symptoms. The clinical manifestations of ODS include weakness, dysarthria, dysphagia, spasticity, and altered levels of consciousness, often resulting in severe morbidity and, in some cases, mortality [5].

Hyponatremia can further be broken down into acute vs chronic hyponatremia with a cut-off of 48 hours duration [6]. This differentiation is a key variant in the management of hyponatremia. In chronic hyponatremia, given the slow progression, the brain helps reduce swelling by allowing electrolytes and organic solutes to exit its cells, which leads to water loss [7]. This process helps alleviate symptoms and reduce swelling, but it also increases the risk of osmotic demyelination syndrome due to the reintroduction of serum sodium [6]. Thus, the correction of hyponatremia, especially in chronic cases, must be approached with precision to minimize the risks of ODS. In cases of overcorrection, therapeutic measures, such as the administration of desmopressin (DDAVP) and infusion of hypotonic fluids like dextrose 5% water (D5W), are used to stabilize and reverse excessive sodium elevation [1]. Such measures in acute hyponatremia are not necessary and will be discussed further.

This report details the case of a 69-year-old male with severe hyponatremia secondary to psychogenic polydipsia, highlighting the pathophysiological mechanisms, complications of sodium dysregulation, and the importance of adhering to controlled correction protocols. The case underscores the complexities involved in managing this condition, emphasizing the need for multidisciplinary collaboration and adherence to established guidelines to optimize patient outcomes.

## Case presentation

**Figure 1:**
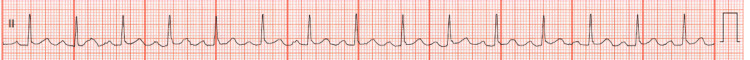
ECG taken upon ED arrival showing PR prolongment; otherwise unremarkable

**Figure 2:**
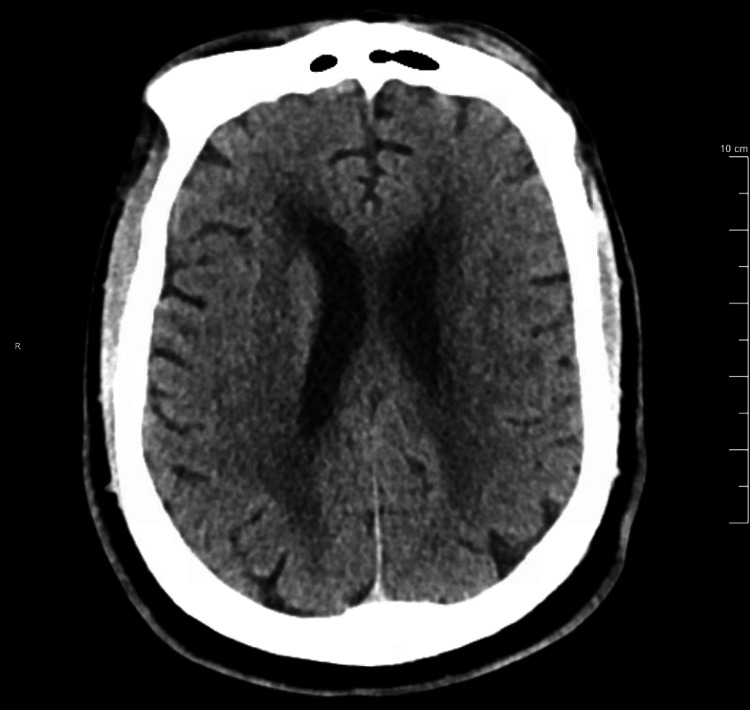
Computed tomography (CT) scan without contrast of head showing no acute process

Our patient is a 69-year-old male with a history of hypertension, osteoarthritis, schizophrenia, and chronic psychogenic polydipsia who presented to the emergency department via ambulance after sustaining a fall. As part of the initial ED workup, the patient had radiographs of his right knee and chest, an ECG, and a CT of the head without contrast; all were grossly unremarkable (Figures 1-2). Additionally, the initial workup revealed severe hyponatremia (serum sodium: 115 mmol/L) accompanied by symptoms of weakness and lethargy, possibly factors that contributed to the fall. Other laboratory findings included severely dilute urine with a urine osmolality of 78 mOsm/kg (normal: >300 mOsm/kg), low renal sodium excretion indicated by a urine sodium level of <20 mmol/L (normal: >40 mmol/L), and a hypo-osmolar state reflected by a serum osmolality of 243 mOsm/kg (normal: 275-295 mOsm/kg). On physical examination, the patient appeared euvolemic, with no signs of peripheral edema, jugular venous distension, or orthostatic hypotension.

Given the patient’s history of recurrent psychogenic polydipsia, a nephrology consult was placed. At the time of the consult, the patient had already received 1 liter of lactated Ringer’s in the emergency department. Repeat blood chemistries revealed serum sodium of 120 mmol/L two hours after presentation, raising concerns for osmotic demyelination syndrome (ODS). Dextrose 5% in water (D5W) was infused at 200mL/hr with plans to follow serum chemistries closely. Despite the infusion of D5W, the patient's serum sodium rose to 129 mmol/L within seven hours of presentation. At this time, a single 2 mcg dose of desmopressin (DDAVP) was given via IV with plans to continue the D5W drip at a rate of 200 mL/hr. An hour later, the sodium was 126 mmol/L, with subsequent hourly testing indicating a sodium value of 124 mmol/L. Sodium levels were gradually normalized 24 hours after the discontinuation of D5W and without further complications. Table 1 provides a detailed report of the patient's laboratory values throughout the hospital course. The patient was eventually discharged after achieving normonatremia.

**Table 1:** Patient's laboratory values throughout the hospital course

Test	Result	Reference Range	Interpretation
Initial Serum Sodium	115 mmol/L	135–145 mmol/L	Severe hyponatremia
Initial Serum Osmolality	243 mOsm/kg	275–295 mOsm/kg	Hypo-osmolar state
Initial Urine Sodium	<20 mmol/L	>40 mmol/L	Low renal sodium excretion
Initial Urine Osmolality	78 mOsm/kg	>300 mOsm/kg	Severely dilute urine
Repeat Serum Sodium (2 hrs post-presentation)	120 mmol/L	135–145 mmol/L	Rising sodium, risk of ODS
Serum Sodium (7 hrs post-presentation)	129 mmol/L	135–145 mmol/L	Rapid correction of sodium
Serum Sodium (8 hrs post-presentation, 1 hr post-DDAVP)	126 mmol/L	135–145 mmol/L	Partial reversal of sodium correction

## Discussion

This case of a 69-year-old male with severe hyponatremia highlights the importance of carefully correcting sodium to prevent ODS. Specifically, this patient’s serum sodium rose from 115 mmol/L to 129 mmol/L, an increase of 14 mmol/L within 7 hours. This surpasses the recommended correction threshold, putting this patient at risk for ODS. This case report aims to highlight the appropriate correction of hyponatremia in a patient with psychogenic polydipsia.

Psychogenic polydipsia, common in patients with schizophrenia, results from an excessive free water load that suppresses ADH while overwhelming renal dilutional capacity [1]. The estimated prevalence of psychogenic polydipsia in patients with schizophrenia specifically is 11-20% [4]. Thus, schizophrenia patients are particularly vulnerable to rapid sodium shifts and their associated neurological risks.

Treatment protocols for both chronic and acute hyponatremia must take into account several factors, including but not limited to the presence of altered central nervous function, the condition's acuity, and its severity, as indicated by sodium concentration levels [6]. Furthermore, the underlying pathology may provide insight into the most appropriate management approach. Whether acute or chronic, water restriction is indicated in the early phases of treatment, such that fluid intake is less than urine output [6]. For acute hyponatremia, the low serum sodium concentration and risk for cerebral herniation and death prompt urgent treatment [8]. The general approach is a 100-milliliter bolus of 3% saline over 10 minutes for patients manifesting with symptoms [8]. For asymptomatic patients, a 50-milliliter bolus of 3% hypertonic saline infused over 10 minutes can be utilized [8]. On the other hand, chronic hyponatremia, while more likely to be asymptomatic due to cerebral adaptation, has three varying treatment protocols as a result of the various extracellular fluid classification statuses [8]. In either case, however, severe symptoms warrant a 100-milliliter bolus of 3% saline infused over 10 minutes [8]. Chronic hypovolemic hyponatremia is treated with 0.9% saline volume replacement, while hypervolemic hyponatremia is treated with fluid and salt restriction, diuresis, and possibly tolvaptan [8]. Similarly, euvolemic hyponatremia is treated with fluid restriction and followed by tolvaptan, urea, or hypertonic saline, if necessitated as second-line treatment options [8]. It is important to note that tolvaptan is a selective arginine vasopressin (AVP) V2 receptor antagonist that blocks AVP binding and, therefore, promotes free water excretion [9]. Urea is an osmotic agent that increases free water excretion and decreases urinary sodium excretion [8].

General guidelines to replenish sodium recommend an initial increase of 4-6 mmol/L in 24 hours, with a maximum correction of 8 mmol/L per day [1]. Overly rapid correction of hyponatremia, while controversial, is determined to be plasma sodium correction greater than 10-12 mEq/L (milliequivalents per liter) in the first 24 hours, greater than 18 mEq/L in the first 48 hours, and greater than 8 mEq/L in any 24-hour period. It is important to clarify, as both units are used throughout this discussion, that one mmol of sodium is equivalent to one mEq [10]. Due to the significant risks associated with overcorrection, the plasma sodium should be therapeutically reduced to below the correction limit [11]. Desmopressin and D5W are two agents commonly used to reduce the plasma sodium concentration, as they halt further water diuresis and dilute the plasma sodium to mitigate the rapid overcorrection of hyponatremia, respectively [11]. Specifically, desmopressin may be infused at a dose of 2-4 mcg every six to eight hours, and D5W may be given intravenously at a rate of 3 ml/kg per hour [11]. In this case, our patient was given a single 2 mcg dose of DDAP to suppress free water clearance, resulting in a partial reversal of sodium overcorrection. Once the desired sodium levels are achieved, adjustments may be made to these agents to prevent further correction. In the presented case, the emergency administration of 1 liter of lactated Ringer’s led to a 5 mmol/L increase within 2 hours, prompting a nephrology consult. Despite an infusion of D5W at 200 mL/hr, sodium levels rose to 129 mmol/L within 7 hours, exceeding the safety threshold for ODS risk. To counteract this rapid overcorrection and prevent ODS, a single 2 mcg dose of DDAVP was administered, successfully stabilizing sodium levels and allowing for gradual normalization without neurological complications.

This case emphasizes the delicate balance required in sodium repletion, particularly in patients with chronic hyponatremia and underlying psychiatric conditions, where close monitoring and early intervention with D5W and DDAVP can prevent life-threatening demyelination.

## Conclusions

This case underscores the challenges of managing hyponatremia in patients with psychogenic polydipsia, particularly in patients with underlying psychiatric conditions. The delicate interactions between excessive water intake, renal excretory limitations, and the risks of overcorrection emphasize the importance of a measured approach to the treatment of hyponatremia. Prompt sodium correction is essential to prevent life-threatening complications, such as cerebral edema, however, rapid overcorrection can pose the risk of osmotic demyelination syndrome. Gradual correction protocols tailored to individual presentations can prevent complications like ODS. This case highlights the importance of identifying acute from chronic hyponatremia as a means to develop the appropriate treatment plan. The adherence to established correction protocols and utilizing interventions such as desmopressin and D5W to mitigate serum sodium overcorrection risks provides physicians with the tools necessary to assure patient safety. Ultimately, the successful management of this patient’s condition reinforces the need for multidisciplinary collaboration, vigilant monitoring, and individualized treatment strategies to optimize outcomes and prevent severe neurological dysfunction. Future efforts should be made to develop standardized, evidence-based guidelines for managing hyponatremia in complex psychiatric populations, ensuring both safety and consistency in care delivery.

## References

[1] Verbalis JG, Goldsmith SR, Greenberg A, Korzelius C, Schrier RW, Sterns RH, Thompson CJ (2013). Diagnosis, evaluation, and treatment of hyponatremia: expert panel recommendations. Am J Med.

[2] Evenson RC, Jos CJ, Mallya AR (1987). Prevalence of polydipsia among public psychiatric patients. Psychol Rep.

[3] Dundas B, Harris M, Narasimhan M (2007). Psychogenic polydipsia review: etiology, differential, and treatment. Curr Psychiatry Rep.

[4] de Leon J, Verghese C, Tracy JI (1994). Polydipsia and water intoxication in psychiatric patients: a review of the epidemiological literature. Biol Psychiatry.

[5] Lambeck J, Hieber M, Dreßing A, Niesen WD (2019). Central pontine myelinosis and osmotic demyelination syndrome. Dtsch Arztebl Int.

[6] Kheetan M, Ogu I, Shapiro JI, Khitan ZJ (2021). Acute and chronic hyponatremia. Front Med (Lausanne).

[7] Giuliani C, Peri A (2014). Effects of hyponatremia on the brain. J Clin Med.

[8] Lawless SJ, Thompson C, Garrahy A (2022). The management of acute and chronic hyponatraemia. Ther Adv Endocrinol Metab.

[9] Dixon MB, Lien YH (2008). Tolvaptan and its potential in the treatment of hyponatremia. Ther Clin Risk Manag.

[10] Hanna RM, Yang WT, Lopez EA, Riad JN, Wilson J (2016). The utility and accuracy of four equations in predicting sodium levels in dysnatremic patients. Clin Kidney J.

[11] Rondon-Berrios H (2020). Therapeutic relowering of plasma sodium after overly rapid correction of hyponatremia. What is the evidence?. Clin J Am Soc Nephrol.

